# Multivariate Analysis of the Determinants of Total Mortality in the European Union with Focus on Fat Intake, Diabetes, Myocardial Infarction, Life Expectancy, and Preventable Mortality: A Panel Data Fixed-Effects Panel Data Model Approach

**DOI:** 10.3390/jcdd11100328

**Published:** 2024-10-15

**Authors:** Silviu Marcel Stanciu, Emilia Rusu, Mariana Jinga, Cosmin Gabriel Ursu, Rares Ioan Stanciu, Daniela Miricescu, Valentin Marian Antohi, Elena Barbu

**Affiliations:** 1 Department of Internal Medicine and Gastroenterology, Carol Davila University of Medicine and Pharmacy, Central Military Emergency University Hospital, 050474 Bucharest, Romania; silviu.stanciu@umfcd.ro (S.M.S.); mariana.jinga@umfcd.ro (M.J.); 2 Department of Diabetology, Carol Davila University of Medicine and Pharmacy, Malaxa Clinical Hospital, 050474 Bucharest, Romania; 3 Faculty of Medicine, Carol Davila University of Medicine and Pharmacy, 050474 Bucharest, Romania; cosmin.d.v.ursu@stud.umfcd.ro (C.G.U.); rares-ioan.stanciu2022@stud.umfcd.ro (R.I.S.); 4 Discipline of Biochemistry, Faculty of Dentistry, Carol Davila University of Medicine and Pharmacy, 8 Eroii Sanitari Blvd, 050474 Bucharest, Romania; daniela.miricescu@umfcd.ro; 5 Department of Business Administration, Dunarea de Jos University, 800008 Galati, Romania; 6 Department of Cardiology, Carol Davila University of Medicine and Pharmacy, Elias Hospital, 050474 Bucharest, Romania

**Keywords:** diabetes, fat intake, health indicators, life expectancy, mortality rate, myocardial infarction, panel data analysis

## Abstract

Cardiovascular disease is the leading cause of death in the European Union (EU), and while the mortality rates of diabetes, myocardial infarction, and the total fat intake have been extensively studied, we believe that understanding the interaction between such closely correlated determinants is crucial to the development of effective health policies in the EU. Our paper’s novelty is represented by the econometric modelling, and its ability to capture both temporal and unit variations. The research methodology consists of using a panel data model with fixed effects for the 27 EU member states over the period 2010–2021. The results of the study show that the standardized mortality rate for deaths preventable by prevention and treatment and diabetes-related mortality are significant predictors of total mortality in the EU. The standardized mortality rate for deaths preventable by prevention and treatment had a significant positive impact, suggesting that improved preventive and therapeutic interventions can significantly reduce total mortality. Diabetes-associated mortality also showed a strong positive correlation with total mortality, emphasizing the need for effective diabetes management and prevention strategies. These results are useful for the formulation of public health strategies aimed at improving life expectancy and reducing the burden of chronic diseases.

## 1. Introduction

Globally, non-communicable diseases (NCDs), such as cardiovascular disease (CVD), diabetes, and cancer, are the leading causes of death, accounting for over 74% of all deaths worldwide [1]. The prevalence of CVD is expected to increase due to an ageing population, urbanization, sedentary lifestyles, and an increase in cardiometabolic risk factors such as obesity, high blood pressure, and type 2 diabetes mellitus.

A diet high in saturated fat, diabetes, and myocardial infarction all significantly influence mortality. Understanding the interaction between these factors and their impact on the overall risk of death is essential. From a simplistic perspective, the clinician is aware that high saturated fat intake is linked to higher mortality and adverse effects on cardiovascular and metabolic health. Estimates suggest that more than 50% of the global population will be overweight or obese by 2035 unless adequate prevention and treatment measures are taken [2]. Excessive consumption of saturated fats adversely affects health and may increase mortality through various mechanisms, such as the development of atherogenic dyslipidemia, intensification of atherosclerotic processes, increased insulin resistance, chronic inflammation, and obesity. The Seven Countries Study, the first multinational epidemiologic study, analyzed the interactions between lifestyle, diet, coronary heart disease, and stroke in diverse populations from different world regions. Results show that countries with high saturated fat consumption have higher rates of death from cardiovascular disease. For example, in Finland, cardiovascular disease mortality was significantly higher compared to Japan, where saturated fat consumption is lower [3]. Atherogenic dyslipidemia, a component of the metabolic syndrome, refers to a lipid profile characterized by elevated triglycerides, small and dense particles of LDL-C and low HDL. This lipid profile is commonly seen in people with glycoregulation abnormalities and contributes to an increased risk of atherosclerotic CVD and complications [4].

Diabetes mellitus is a chronic disease with increasing prevalence and profound public health implications. In addition to genetic factors, the main determinants of type 2 diabetes are diet, sedentary lifestyle, and, consequently, obesity [5]. Authors Kaptoge et al. [6] explore the increased prevalence of type 2 diabetes, particularly among young people, and its impact on mortality and life expectancy, using data from two large sources, the Emerging Risk Factors Collaboration (ERFC) and the UK Biobank, comprising 1,515,718 participants from 19 high-income countries, followed up for a total of 23.1 million person-years. The analysis shows that in the US, a 50-year-old with diabetes diagnosed in their 30s dies on average 14 years earlier than someone without diabetes, and in the EU, estimates show similar reductions in life expectancy. The study concludes that each decade of earlier diagnosis of type 2 diabetes is associated with a decrease of about 3–4 years in life expectancy.

Acute myocardial infarction is one of the leading causes of death globally, and its prevalence is influenced by factors such as gender, age, diet, physical activity level, and access to prompt medical intervention. Authors Batya Betesh-Abay et al. [7] evaluate the relationship between survival analysis coefficients and life expectancy in post-acute myocardial infarction patients, using a survival analysis-based methodology to assess the relative risk of risk factors for mortality over a follow-up period of up to 21 years. The study demonstrates that the presence of additional risk factors significantly reduces life expectancy.

Another study by the authors Bucholz et al. [8] analyses differences in life expectancy and potential years of life lost after an acute myocardial infarction by sex and race, using data from the Cooperative Cardiovascular Project with 146,743 patients. The results show that women lost, on average, 10.5% more of their expected life than men, and black patients lost 6.2% more than whites. After adjustment, women lost 7.8% more of their expected life than men, but the racial differences were explained by different clinical presentations and treatments. The study’s conclusion highlights that women and patients of color have a significant disadvantage in post-AMI survival, highlighting the need for secondary prevention measures and equitable care to reduce these disparities.

The analysis of mortality and its determinants has recently become a priority for public health policies in the European Union (EU). Total mortality is a crucial indicator of population health, reflecting the combined impact of socio-economic, environmental, and health factors. In the context of the diversity of EU member states, it is vital to better understand the impact on total mortality of specific highly interrelated variables such as life expectancy, fat intake, diabetes mellitus, and acute myocardial infarction. International studies show a strong correlation between socio-economic factors and mortality rates, highlighting the crucial role of education and income in improving health outcomes [9]. Thus, life expectancy in a given population, which has economic and social determinants, strongly influences morbidity and mortality rates.

Understanding the interaction between closely correlated determinants such as life expectancy, fat intake, diabetes mellitus, and acute myocardial infarction is essential for determining overall mortality rates and crucial for developing effective public health policies in the EU. This research can contribute to the literature by applying fixed-effects panel data analysis, providing a detailed and nuanced perspective on the determinants of total mortality.

The paper’s novelty is represented by the econometric modelling, and the application of the panel data model with fixed effects to capture both temporal and unit (the 27 European countries) variations, providing a detailed and complex picture of the factors influencing mortality. Although there are numerous studies in the literature on the impact of acute myocardial infarction, diabetes mellitus, and fat intake on mortality and common pathogenic mechanisms are well defined, few studies have addressed the combined interaction of these variables using a panel data fixed-effects model.

At the same time, life expectancy at birth is a critical demographic indicator, reflecting the health status of the population and the efficiency of health systems. Life expectancy is an indicator of access to health care, educational attainment, living conditions, nutrition, and other social determinants of health. Countries with higher life expectancy have lower mortality rates from chronic diseases such as cardiovascular disease and cancer due to advanced prevention and treatment. For example, mortality from cardiovascular disease is 50% lower in countries where life expectancy at birth is over 80 years compared to those where life expectancy is below 65 years [10]. The INTERHEART study [11], conducted in 52 countries, found that nine risk factors, which can be easily measured and modified, could account for more than 90% of the risk of myocardial infarction worldwide across all regions and major ethnic groups. As well as emphasizing the increased predictive value of smoking and circulating cholesterol for the development of myocardial infarction, the study shows that access to medical care and educational attainment influence mortality. Patients in countries with higher income and better life expectancy at birth had lower mortality from heart attack compared to those in low- and middle-income countries. Preventable mortality reflects deaths that could have been avoided by appropriate medical or public interventions. Its impact on global mortality is considerable, as many global deaths are the result of diseases and conditions that can be prevented or effectively treated. Mortality rates from preventable causes such as metabolic diseases reflect the effectiveness of public health systems and preventive interventions.

## 2. Research Methodology

In order to determine the direct, indirect, and causal relationships between the variables, providing a complete picture of the factors influencing the causes of death—standardized total mortality rate (deaths per 100,000 population), we collected the database and analyzed data on the following specific indicators from the reports of 25 European countries selected for the study. This information was extracted from public sources accessible through the OECD.Stat platform [12], covering the period 2010 to 2021, as shown in Table 1.

By using panel data analysis, it can be determined whether the explanatory variables (standardized mortality rate for acute myocardial infarction (deaths per 100,000 population), total fat intake or total fat consumption, standardized mortality rate avoidable by prevention and treatment (deaths per 100,000 population), standardized mortality rate for diabetes mellitus (deaths per 100,000 population), and life expectancy at birth) have a significant impact on the dependent variable (standardized total mortality rate). This type of analysis allows for the capture of both temporal variation and variation between units (e.g., the 27 EU member states), providing a more complete picture of the factors that may influence mortality.

The choice of diseases such as acute myocardial infarction and diabetes mellitus as explanatory variables in the analysis of total mortality is well founded due to their epidemiologic relevance, significant economic and social impact, interaction with other risk factors, and availability of data. The study of these two diseases may provide valuable insights for public health policies and interventions aimed at reducing mortality and improving population health. The choice of the independent variable total fat intake as a non-medical determinant of health is well justified due to its direct link with cardiovascular disease and obesity, its role as an indicator of diet quality, its modifiability through public health interventions, and the availability of relevant data. Studying this variable may provide valuable insights for the development of health policies and nutritional interventions aimed at improving population health and reducing mortality associated with unhealthy fat intake. 

The standardized rate of mortality avoidable by prevention and treatment as an independent variable in the analysis of total mortality is well justified because of its direct relevance for public health policies, its link with overall mortality, its impact on the effectiveness of health interventions, its usefulness in econometric analyses, and its interaction with other factors of interest. Studying this variable can provide valuable information for improving health systems and reducing avoidable mortality, thus contributing to increasing life expectancy and improving population health. The use of the independent variable life expectancy at birth as an independent variable in the analysis of total mortality is also well justified because of its demographic and public health relevance, its inverse relationship with mortality, its impact on health policies, its interaction with other determinants of health, and its usefulness in econometric analyses. Studying this variable can provide valuable information for the development and evaluation of health policies, thus contributing to improving the health and well-being of the population.

The use of panel data analysis in the study of standardized all-cause mortality rates (ALLCD) and independent variables (AMCINF, TFATS, AVMORT, DIABMEL, LIFEEXP) is well justified because of its ability to capture individual and temporal heterogeneity, increase the number of observations and the robustness of estimates, and detect causal relationships and complex interactions. These methodological advantages contribute to a more detailed understanding of the factors influencing total mortality and to the development of more effective health policies.

The fixed-effects panel model equation, according to the indicators presented in the Table 1, is presented below:

$${\mathrm{ALLCD}}_{\mathrm{it}}=1735.049+0.342*{\mathrm{AMCINF}}_{\mathrm{it}}+0.134*{\mathrm{TFASTS}}_{\mathrm{it}}+1.654*{\mathrm{AVMORT}}_{\mathrm{it}}+0.984*{\mathrm{DIABMEL}}_{\mathrm{it}}-15.833*{\mathrm{LIFEEXP}}_{\mathrm{it}}+u_{\mathrm{it}}+\varepsilon_{\mathrm{it}}$$

where

- ${\mathrm{ALLCD}}_{\mathrm{it}}$ is the dependent variable for decision unit i at time t;
- ${\mathrm{AMCINF}}_{\mathrm{it}},\;{\mathrm{TFASTS}}_{\mathrm{it}},\;{\mathrm{AVMORT}}_{\mathrm{it}},\;{\mathrm{DIABMEL}}_{\mathrm{it}},\;{\mathrm{LIFEEXP}}_{\mathrm{it}}$ are the independent variables for decision unit i at time t;
- $u_{\mathrm{it}}$ is the unobserved individual effect (fixed effect);
- $\varepsilon_{\mathrm{it}}$ is the error term for decision unit i at time t.

The use of the fixed-effects panel model for the analysis of the standardized total mortality rate and associated independent variables offers a number of analytical and application advantages. These include controlling for unobserved heterogeneity, assessing short- and long-term effects, increasing the precision of estimates, detecting causal relationships, and analyzing interactions. All these advantages contribute to a better understanding of factors influencing total mortality, providing information to develop effective public health policies.

Based on the data in Table 1, we formulated working hypotheses involving interactions between multiple variables to assess the relationships and combined effects of the independent variables on the all-standardized total mortality rate (ALLCD). 

Acute myocardial infarction is one of the leading causes of death globally, with a direct impact on total mortality. Studies have shown that acute myocardial infarction contributes substantially to total mortality and is responsible for a significant number of deaths in the population. Myocardial infarction is a life-threatening coronary heart disease characterized by sudden cardiac death. In a recent study, the authors Salari et al. [13] conducted a comprehensive analysis using various databases to identify complications of myocardial infarction and designed prevention programs. Twenty-two eligible studies were included, with a sample size of 29,826,717 people (<60 y). The results of the study show that the overall prevalence of myocardial infarction in people under 60 y was 3.8%, while in those over 60 y it was detected at 9.5%. The study concludes that because of the accelerated rate of myocardial infarction prevalence in older ages, accurate patient attention to myocardial infarction complications is significant and preventive planning and safe treatment methods are essential. 

Studies have shown that cardiovascular disease is one of the leading causes of death globally, and acute myocardial infarction is a major contributor to this statistic [14].

However, the relationship between the standardized mortality rate for acute myocardial infarction and the standardized total mortality rate is complex and may be influenced by various risk factors such as total fat intake (TFATS) and life expectancy at birth (LIFEEXP). Studies show that high fat intake is associated with an increased risk of cardiovascular disease, which may indirectly influence total mortality [15,16]. Life expectancy at birth is also an indicator that reflects both health conditions and the effectiveness of the healthcare system [17]. 

The standardized mortality rate for acute myocardial infarction is a key indicator used to assess the impact of this disease on a population. It measures the number of deaths due to acute myocardial infarction, adjusted to remove the effects of demographic variables such as age and gender to allow fair comparisons between different populations and time periods. Thus, we can formulate the following research hypothesis:

**Hypothesis** **1.** *The standardized mortality rate for acute myocardial infarction (AMCINF) indirectly influences the standardized rate of total mortality (ALLCD) through its impact on the standardized rate of mortality that can be avoided by prevention and treatment (AVMORT).*

Deaths that can be avoided by preventive measures and appropriate treatment are those that can be prevented by effective medical interventions and public health policies. Preventive interventions and appropriate treatment can significantly reduce the number of avoidable deaths and, thus, have a direct impact on reducing overall mortality [18]. Health policies that emphasize prevention and effective treatment are key to reducing mortality [19]. An effective health system is able to significantly reduce avoidable mortality by early identification of conditions and implementation of effective interventions. Thus, the decrease in the avoidable mortality rate has a direct impact on the total mortality rate, reflecting the ability of a health system to protect and improve the health of the population, and the following research hypothesis can be proposed:

**Hypothesis** **2.** *The standardized rate of preventable mortality avoidable by prevention and treatment (AVMORT) strongly influences the standardized rate of total mortality (ALLCD).*

Diabetes mellitus is a chronic condition that, left untreated, can lead to severe complications and death. Diabetes has a high prevalence and is associated with numerous complications that increase the risk of death [20]. Proper management of diabetes mellitus can significantly reduce the mortality rate associated with the disease [21]. The effective management of diabetes mellitus through blood glucose monitoring, dietary interventions, physical activity, and appropriate drug therapies can significantly reduce associated complications and, therefore, mortality from the disease. Understanding the relationship between diabetes-related mortality and overall mortality is essential for the development of public health strategies to improve the prevention and treatment of diabetes mellitus. We propose the following research hypothesis:

**Hypothesis** **3.** *The standardized mortality rate for diabetes mellitus (DIABMEL) is a significant predictor of the standardized all-cause mortality rate (ALLCD).*

Dietary fat intake is a major risk factor for cardiovascular disease, including acute myocardial infarction [22]. A diet high in saturated fat can lead to the build-up of atherosclerotic plaques in the arteries, increasing the risk of acute myocardial infarction. Reducing the intake of unhealthy fats may help to reduce the incidence of myocardial infarction and, therefore, the associated mortality [23]. Total fat intake (TFATS) is an essential indicator for assessing cardiovascular risk in a population. Measuring grams of fat consumed per capita per day provides valuable information about dietary habits and potential risks to heart health. In the context of public health, it is important to analyze the link between fat intake and the incidence of acute myocardial infarction in order to develop dietary interventions that reduce the risk of these conditions and, therefore, contribute to improving cardiovascular health, allow us to formulate the following research hypothesis:

**Hypothesis** **4.** *Total fat intake (TFATS) influences the standardized mortality rate for acute myocardial infarction (AMCINF).*

Life expectancy at birth reflects the estimated average life expectancy of a population under current socio-economic and health conditions. Studies show that a higher life expectancy indicates an efficient and accessible health system, with preventive measures and appropriate treatment that can reduce avoidable deaths [24,25]. Countries with high life expectancy tend to have effective health policies that contribute to lower avoidable mortality. Life expectancy at birth is a key demographic indicator that reflects the average lifespan of a population, emphasizing the impact of socio-economic conditions, environment, and access to health services. This indicator provides an insight into the overall health of the population and the effectiveness of health systems in preventing and treating disease.

The standardized preventable mortality rate (AVMORT) measures the number of deaths that could be prevented by effective public health interventions and appropriate treatment. A well-structured and accessible health system contributes significantly to reducing these avoidable deaths, which is, thus, reflected in the higher life expectancy. 

Analyzing the relationship between life expectancy and avoidable mortality is important for assessing the performance of health systems and for developing strategies to improve the prevention and treatment of medical conditions. A high life expectancy suggests effective public health management with preventive measures and therapeutic interventions that reduce avoidable mortality, thus we can formulate the following research hypothesis:

**Hypothesis** **5.** *Life expectancy at birth (LIFEEXP) is inversely proportional to the standardized preventable mortality rate (AVMORT).*

## 3. Results

Analyzing the relationship between health indicators and their impact on mortality and life expectancy using the fixed-effects model has the ability to control for unobserved heterogeneity between entities, providing accurate and relevant results. The indicators used are essential for understanding the general health status of the population and the major risk factors.

Table 2 reports the results of the Hausman test, which assesses the consistency and efficiency of fixed- and random-effects models. This statistical test helps determine whether unique errors (*ui*) are correlated with regressors, which has significant implications for the interpretation of our regression results.

The Hausman test results indicate a significant difference in coefficients (Chi^2^ (5) = 124.06, Prob > chi^2^ = 0.0000), which indicates that the null hypothesis is rejected, suggesting that the fixed-effects model is more appropriate for the indicators used. The differences between fixed and random effects coefficients are shown in column (b-B). These differences indicate how much the coefficients vary between the two models. The consistency of the fixed-effects model indicates that it better accounts for unobserved heterogeneity between entities. The Hausman test tests the null hypothesis (H0) that the differences between the coefficients are not systematic. The model helps to accurately capture the impact of different health indicators on mortality rates and life expectancy. 

The Breusch–Pagan LM test of independence is used to check for the presence of correlations between errors (u_i) of different groups (DMU) in the panel regression model. Values obtained chi^2^ (300) = 678.519 and Pr = 0.000.

The Modified Wald test was applied, used to test for the presence of heteroscedasticity obtaining chi^2^ (25) = 1348.08 and Prob > chi^2^ = 0.0000. The associated probability value (0.0000) indicates high significance, which means that there is very strong evidence against the null hypothesis of homoscedasticity. Thus, the null hypothesis is rejected with a very high level of confidence.

The Pesaran test for cross-sectional independence was applied, being used to assess the presence of dependence between cross-sectional units (cross-sectional) within the fixed-effects panel data model. This test is important to detect correlations between different unit errors, which may affect the validity of statistical conclusions. The test results were: Pesaran’s test of cross sectional independence = 3.736, Pr = 0.0002, average absolute value of the off-diagonal elements = 0.363. The probability value associated with the test is very small (0.0002), indicating high significance, therefore, we reject the null hypothesis with very high confidence.

Table 3 shows the results of the fixed-effects model.

The panel data fixed-effects panel data model demonstrated robust explanatory power, with an R-squared of 0.8133 for within-group variation and 0.9337 overall. This indicates that the model explains most of the variation in total mortality rate, suggesting that the selected independent variables (AMCINF, TFATS, AVMORT, DIABMEL, and LIFEEXP) are relevant and important factors in determining total mortality in European states.

The significant coefficients of AVMORT, DIABMEL, and LIFEEXP suggest that these variables have a significant impact on total mortality. For example, AVMORT, which measures the rate of avoidable mortality through prevention and treatment, has a significant positive coefficient, indicating that improved preventive and therapeutic interventions can reduce total mortality. This highlights the importance of investing in prevention and treatment in public health policies.

Similarly, the positive and significant coefficient of the DIABMEL variable suggests that diabetes mellitus is a major predictor of total mortality. This highlights the need for diabetes management and prevention programs that can significantly reduce the mortality rate associated with diabetes.

The significant negative coefficient of the LIFEEXP variable indicates an inverse relationship between life expectancy at birth and the total mortality rate. This suggests that improving general health conditions and increasing life expectancy contribute to reducing total mortality.

The indicator acute myocardial infarction standardized mortality rate (AMCINF) has a positive coefficient, but it is not statistically significant (*p* = 0.112). However, we cannot conclude that this indicator directly influences the standardized all-cause mortality rate (ALLCD); the positive coefficient suggests that an increase in this indicator could be associated with an increase in the standardized all-cause mortality rate in ALLCD. Even if the coefficient is not statistically significant, this does not necessarily mean that there is no practical or clinical relationship between the AMCINF and ALLCD indicators. Although the AMCINF indicator is not directly significant, the literature suggests that prevention and treatment of myocardial infarction may reduce avoidable mortality (AVMORT) [26], and the AVMORT indicator has a highly significant and positive coefficient on the standardized total ALLCD mortality rate, thus interventions to reduce AMCINF may indirectly reduce ALLCD by decreasing AVMORT. Hypothesis 1 is validated by both the data analysis in Table 3 and the results from the literature. Effective interventions on acute myocardial infarction (AMCINF) can reduce avoidable mortality (AVMORT), which has a significant impact on total mortality rate (ALLCD). Therefore, reducing AMCINF may have an indirect but significant effect on reducing ALLCD, supported by multiple studies in the literature.

According to Rohani et al. [27], cardiovascular disease, including acute myocardial infarction, accounts for a significant proportion of avoidable mortality. Effective interventions can significantly reduce mortality through medical treatments and lifestyle changes. Another study by Bhatt et al. [28] has shown that treatments for myocardial infarction, such as rapid aspirin, thrombolytics, and angioplasty, have significantly reduced mortality in recent decades.

The preventable avoidable mortality rate through prevention and treatment (AVMORT) had a coefficient of 1.654, which is highly significant (*p* < 0.001), indicating that an increase in the preventable avoidable mortality rate through prevention and treatment (AVMORT) is associated with a significant increase in the total mortality rate (ALLCD). This emphasizes the importance of preventive measures and effective treatments in reducing total mortality. The very small *p* value (*p* < 0.001) indicates that there is a highly significant relationship between AVMORT and ALLCD. Thus, we can reject the null hypothesis that there is no relationship between the two variables. The coefficient of 1.654 suggests that for every 1 unit increase in AVMORT (avoidable deaths per 100,000 population), the total mortality rate (ALLCD) increases by 1.654 units. This significant coefficient reflects the large impact of avoidable mortality on total mortality. These results emphasize that many deaths are avoidable with appropriate prevention and treatment measures. Public health interventions that focus on prevention, e.g., vaccinations, screening for chronic diseases, health education programs, and effective treatment, access to medicines, and management of chronic diseases, are essential to reduce total mortality.

The hypothesis that the avoidable mortality rate from prevention and treatment (AVMORT) strongly influences the all-cause mortality rate (ALLCD) is well supported by both our model data and the literature. Reducing AVMORT through effective preventive interventions and treatment is essential for improving population health and reducing total mortality. A significant proportion of deaths worldwide are considered avoidable through effective public health interventions.

This includes the prevention and treatment of non-communicable diseases (e.g., cardiovascular disease, diabetes), which are responsible for a large share of global mortality [29]. According to European studies, primary and secondary prevention, including treatments for hypertension and high cholesterol, have significantly reduced cardiovascular mortality, a major component of the AVMORT indicator [30].

The coefficient of the total fat intake (TFATS) indicator had a low value of 0.134, but with a *p*-value of 0.475, indicating that this indicator does not have a significant direct effect on total mortality (ALLCD). This result suggests that variations in total fat intake do not significantly explain variations in total mortality rate in this model. The coefficient of 0.134 suggests a weak positive relationship, where an increase in fat intake could be associated with an increase in total mortality. However, due to a lack of statistical significance, this effect cannot be considered robust. The health effects of total fat intake may be influenced by the type of fat consumed. Saturated fats may have negative effects, while unsaturated fats, such as fish and vegetable oils, may have beneficial effects on cardiovascular health and mortality. Differences in the effects of dietary fats may be masked in a model that does not differentiate between fat types [31].

The hypothesis that total fat intake (TFATS) influences the total mortality rate (ALLCD) is not validated by the data in our model, given the insignificant coefficient (*p* = 0.475). This may be explained by the fact that the health effects of fat depend on the type of fat consumed rather than total fat intake.

The coefficient of 0.984 for the diabetes mortality rate (DIABMEL) is highly significant (*p* < 0.001), indicating that an increase in the diabetes mortality rate is associated with a significant increase in the total mortality rate (ALLCD), for every 1 unit increase in the diabetes mortality rate (deaths per 100,000 population), the total mortality rate (ALLCD) increases by 0.984 units.

This result suggests that diabetes mellitus is an important predictor of total mortality. Diabetes mellitus is associated with numerous chronic complications, including cardiovascular disease, renal failure, and amputations, all of which contribute to increased mortality. Ineffective diabetes management can lead to acute complications, such as diabetic ketoacidosis and severe hypoglycemia, which are also lethal.

The hypothesis that the mortality rate for diabetes mellitus (DIABMEL) is a significant predictor of the all-cause mortality rate (ALLCD) is well supported by both our model data and the literature. Numerous studies show that diabetes mellitus is associated with an increased risk of all-cause mortality due to the multiple complications associated with the disease [32,33,34]. Diabetes mellitus contributes significantly to overall mortality through associated chronic and acute complications, and appropriate management of this disease is essential to reduce mortality.

The coefficient value of −15.833 for the life expectancy at birth (LIFEEXP) indicator is highly significant (*p* < 0.001), indicating that an increase in life expectancy at birth is associated with a significant decrease in the total mortality rate (ALLCD). This result suggests that improvements in life expectancy reflect improvements in overall health and reduced mortality. The hypothesis that life expectancy at birth (LIFEEXP) is inversely proportional to the total mortality rate (ALLCD) is well supported by both the data in our model and the literature. Increases in life expectancy reflect improvements in general health, living conditions, and public health policies, all of which contribute to a reduction in total mortality. Higher life expectancy is usually associated with improvements in living conditions, access to health care, nutrition, education, and socio-economic factors. Countries with higher life expectancy tend to have more efficient health systems and well-implemented public health policies, which contribute to reduced mortality. This inverse relationship can be explained by the fact that, in societies with low avoidable mortality rates, preventive measures and medical intervention play a central role in protecting the health of the population, which directly contributes to prolonging average life expectancy. Life expectancy is also a reflection of a multitude of interrelated factors, such as economic conditions, the level of education, the lifestyle of individuals, and the quality of the environment, all of which contribute to an environment conducive to longer and healthier lives. At the same time, education plays an important role in the adoption of healthy behaviors, such as reducing tobacco and alcohol consumption or preventing chronic diseases through balanced nutrition and physical activity. This is amplified by a robust health infrastructure and government policies that prioritize prevention and equitable access to high-quality health care. Thus, the standardized avoidable mortality rate becomes an important indicator quantifying the success of health systems and public policies in reducing preventable premature deaths. The negative correlation with life expectancy highlights that a decrease in life expectancy is associated with an overall improvement in the health of the population and, hence, an increase in life expectancy. This dynamic underlines the importance of pro-active public health measures in reducing health inequalities and prolonging the lives of individuals. Therefore, life expectancy at birth can be considered as an important covariate in the analysis of factors influencing public health, providing a global perspective on the complex relationships between education, economic conditions, environment, access to health care, and mortality.

The phrase “Standardized mortality rate avoidable by prevention and treatment” refers to the proportion of preventable deaths in a population with prompt and efficient public health measures and medical care. Understanding avoidable mortality involves breaking down the concept into two categories: preventable mortality, which refers to deaths that could have been averted through primary prevention strategies such as reducing risk factors; and treatable mortality, which includes deaths that could have been prevented with medical interventions proven to be effective in reducing mortality. 

The measurement is particularly important for chronic conditions like type 2 diabetes and cardiovascular diseases. These conditions have a significant impact on mortality rates. The underlying causes are complex and include ineffective or inadequate preventive measures, as well as patients who remain untreated or inadequately treated due to lack of awareness, noncompliance, or underuse of certain treatments that have been proven to reduce mortality rates.

In regarding to preventable measurements, consistent physical activity and diet are crucial in the prevention and management of T2D and CVD. A high intake of saturated fats is associated with a greater risk of CVD and impaired glycemic control. The intake of total fats is important, as both saturated and unsaturated fats have significant impacts on health and mortality. Saturated fats, mainly found in animal products and some tropical oils, are linked to higher levels of low-density lipoprotein (LDL) cholesterol, atherosclerosis, and CVD [35]. On the other hand, unsaturated fats, such as monounsaturated and polyunsaturated fats, lower LDL cholesterol and increase high-density lipoprotein (HDL) cholesterol, providing heart-protective benefits and contra balance the saturated intake fat outcomes [36,37]. Reducing saturated fats is important, but it is also beneficial to combine unsaturated fats with a diet abundant in antioxidants. Antioxidants, found in fruits, vegetables, nuts, and seeds, combat free radicals and decrease oxidative stress, thus preventing cell damage and reducing the risk of chronic diseases, in particularly metabolic diseases such as diabetes or CVD. Hence, a diet rich in antioxidants can counteract the negative effects of saturated fats. Studies indicate that polyphenol consumption is associated with a reduced risk of mortality from all causes, primarily due to their capacity to lower oxidative stress, and a reduction in total free fatty acids (FFAs) modulates inflammatory pathways and improves endothelial function [38]. These mechanisms play a role in decreasing the risk of cardiovascular diseases and other chronic conditions, including neoplasia. Therefore, maintaining a balance between saturated and unsaturated fat intake, coupled with a diet rich in antioxidants, is advisable for lowering the risk of heart diseases and enhancing metabolic health.

Regarding treatment measurements, only specific treatments have been demonstrated to reduce the mortality rate in T2D and CVD. GLP-1 receptor agonists (GLP-1RA) and SGLT2 inhibitors (SGLT2i) both reduce blood glucose levels and cardiovascular events, thereby decreasing overall mortality. They have become leading classes in the management of type 2 diabetes, showing superior efficacy in reducing mortality rates compared to other antidiabetic drugs. These drugs also reduce cardiovascular mortality and heart failure hospitalization, with mortality reductions of up to 30% in high-risk populations [39,40,41]. The use GLP-1 receptor agonists, including liraglutide, semaglutide, and dulaglutide to boost insulin secretion dependent on glucose, diminish glucagon levels, enhance satiety, and improve weight loss. Beyond glycemic control, GLP-1RAs have shown cardiovascular benefits. Research from the LEADER and SUSTAIN-6 trials indicates substantial decreases in major adverse cardiovascular events and cardiovascular mortality in patients receiving GLP-1Ras [42,43,44]. Large-scale clinical trials have consistently shown that SGLT2 inhibitors significantly lower the risk of cardiovascular events in individuals with type 2 diabetes. For instance, the EMPA-REG OUTCOME trial revealed a 38% reduction in cardiovascular death risk with empagliflozin [45] and the DAPA-HF trial indicated that dapagliflozin decreased the risk of heart failure progression or cardiovascular death by 26% in heart failure patients, regardless of their diabetes status [46]. SGLT2 inhibitors also proved renal protection by improving hemodynamics and reducing glomerular hyperfiltration, thereby slowing the progression of diabetic kidney disease and indirectly reducing cardiovascular and all-cause mortality rates [47,48]. Compared to traditional diabetes medications like sulfonylureas and insulin, GLP-1 receptor agonists GLP-1RAs and SGLT2 inhibitors demonstrate stronger evidence for reducing mortality. While older therapies mainly target glycemic control, they lack significant evidence of decreasing mortality rates or cardiovascular risk, though the type of treatment used is important in reducing mortality. 

A key issue in reducing avoidable mortality is the proportion of people with T2D who remain untreated or under-treated. Globally, approximately 50% of people with T2D are undiagnosed, with rates varying by region. In developed countries, the undiagnosed rates range from 10% to 30%, while in developing countries, they can be even higher. Moreover, among those diagnosed, 20–40% do not achieve target control of glucose, blood pressure, or lipids, resulting in preventable complications and deaths [49,50,51]. Several factors contribute to this issue, including a lack of awareness due to non-specific or absent early symptoms; limited access to care due to economic and geographic barriers, particularly in low- and middle-income countries; healthcare system inefficiencies such as inadequate patient follow-up; high costs of effective medications like SGLT2 inhibitors and GLP-1 receptor agonists; and, finally, patient adherence. Side effects, costs, or misunderstandings about the disease’s severity frequently challenge patients’ long-term adherence to treatment. Enhancing prevention, early diagnosis, and effective treatment accessibility can decrease preventable deaths linked to type 2 Diabetes and CVD. Undiagnosed T2D, its regional disparities, and the suboptimal management of diabetes among those diagnosed underscore the importance of addressing the global public health implications of these conditions.

The analysis underlines the importance of public health policies that focus on the prevention and treatment of chronic diseases, as well as on the general improvement in living conditions. By prioritizing these aspects, a significant reduction in the overall mortality rate at the European level can be achieved.

Thus, life expectancy suggests that improving general health conditions and quality of life is critical for reducing total mortality. Investments in health education, health care, and healthy living conditions are important for reducing mortality. These findings emphasize the importance of integrated and coordinated public health measures to effectively address the factors influencing total mortality and to improve the health of the population at European level.

## 4. Discussion

Total mortality is a fundamental indicator of population health and reflects many socio-economic, environmental, and health factors. In the context of the EU, where there is considerable diversity in health policies, economic conditions, and lifestyles, multivariate analysis of the determinants of total mortality is important for effective policy formulation and targeting of resources.

The fixed-effects panel data model is an econometric tool for this analysis because it allows for control for unobserved variables that are constant over time for each unit of analysis but vary between units. This model helps to remove the bias caused by these unobserved variables by providing more precise estimates of the effects of independent variables on total mortality.

In the EU, member states face different health challenges, influenced by factors such as life expectancy, prevalence of chronic diseases, access to health care, and dietary habits. 

Table 4 presents the descriptive statistics of the indicators analyzed in the model.

From the Table on descriptive statistics, it can be observed that the standardized total mortality rate recorded a mean value of 961.44 and a standard deviation of 211.62. The maximum value recorded was 1555.96 in the year 2021 in Bulgaria. At the opposite pole, the lowest recorded value of 670.7 was recorded in 2019 in Spain. Bulgaria has the highest total mortality rate, which may indicate significant public health problems and limited access to quality health care services. In contrast, Spain, with the lowest, may benefit from a more efficient health system and better living conditions.

The standardized mortality rate for acute myocardial infarction shows a tendency of dispersion of the values compared to the calculated average of 44.47, with the average being exceeded by up to five times in the period analyzed at the level of the member states. The maximum value of this indicator, 112.6, was recorded in 2010 in Romania. The minimum value of 13.03 was recorded in 2021 in France. Romania records the highest mortality rate from acute myocardial infarction, suggesting the need for more effective prevention and treatment measures. France, with the lowest value, can serve as a model for good practice in the prevention and treatment of cardiovascular disease.

The total fat intake recorded in the analyzed period 2010–2021 a mean of 135.26 and a standard deviation of 19.51. The highest value was recorded in 2020 in Austria. At the opposite pole, the lowest value (88.2) was recorded in 2014 in Bulgaria. Austria, with the highest value, may indicate a high dietary fat intake, which may contribute to health risks if not accompanied by an active lifestyle. Bulgaria, with the lowest value, may have a lower fat intake, but it is important to assess whether this is optimal for health.

The standardized mortality rate that can be avoided by prevention and treatment recorded an average value of 106.57 during the period under review. The highest value of 532 was recorded in 2010 in Latvia and the lowest value of 135.4 was in 2021 in Italy. States with high AVMORT values need to invest in prevention and treatment. Screening programs, access to treatment, and education campaigns are key to reducing these rates.

In terms of life expectancy at birth in the analyzed period 2010–2021, we observe a standard deviation of 2.98 and a mean value of 79.45. The maximum value of 84 years was recorded in 2019 in Spain and the minimum value of 71.4 years was recorded in 2021 in Bulgaria. Spain, with the highest life expectancy, indicates an efficient healthcare system and good living conditions. Bulgaria, with the lowest life expectancy, reflects great needs to improve public health and living conditions.

Analysis of descriptive statistics reveals significant variations between EU member states in total mortality, cardiovascular disease, fat intake, avoidable mortality, and life expectancy. These disparities highlight the need for tailored policies and interventions to address country specificities. Investments in prevention, treatment, health education, and improved living conditions are essential to reduce mortality and increase life expectancy in the EU.

**Figure 1:**
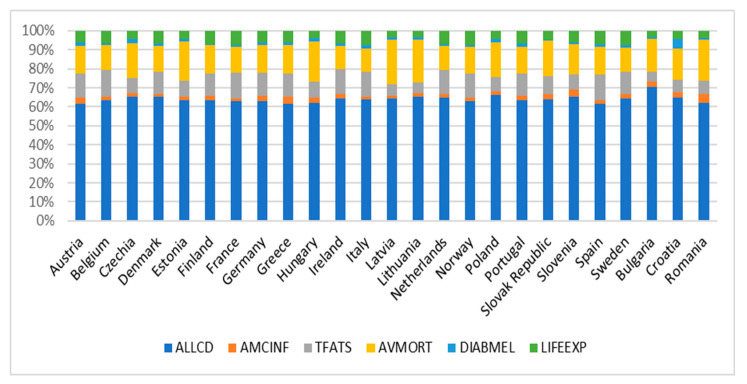
Evolution of health indicators analyzed for EU member states in 2021. Source: Realized by the authors.

Figure 1 shows the evolution of the analyzed indicators in 2021 for the EU member states.

The multivariate analysis highlights the essential role of prevention and treatment measures in reducing total mortality in the EU. It also highlights the importance of an integrated approach to health and lifestyle factors to improve population health and reduce disparities between member states.

Based on the findings from the multivariate analysis of the determinants of total mortality in the EU, we have formulated the following policies to effectively address these determinants and improve public health: prevention and treatment policies for chronic diseases; improved access to modern treatments; policies for the management and prevention of diabetes mellitus; and policies for nutrition and the promotion of healthy lifestyles.

The implementation of these policies contributes significantly to reducing overall mortality in the EU and improving the health of the population. Policies on prevention and treatment, chronic disease management, promotion of healthy lifestyles, and improvement in socio-economic conditions are important to address the diversity and complexity of mortality determinants in EU member states.

One of the major strengths of this study is the use of the standardized mortality rate as a central indicator to assess the causes of death and the impact of risk factors such as acute myocardial infarction, diabetes mellitus, and nutrition. By addressing these variables, the study emphasizes the importance of prevention and treatment interventions in reducing avoidable deaths, highlighting the need for tailored public policies. In addition, the integration of data on life expectancy at birth provides a broad perspective on the health of the population and the efficiency of the health system in EU member states. The use of data on total fat intake also allows for the impact of diet on health to be assessed, providing a sound basis for nutrition and public health policies.

On the other hand, the study has certain limitations that need to be considered, i.e., significant variations between EU member states in terms of health infrastructure and access to modern treatments may influence the results, suggesting that the data do not always reflect a complete and uniform picture.

## 5. Conclusions

The study achieved its main research objective to assess the impact of indicators such as acute myocardial infarction mortality rate, total fat intake, preventable mortality rate by prevention and treatment, diabetes mellitus mortality rate, and life expectancy on total mortality rate in the EU using panel data fixed-effects analysis. The multivariate analysis model provides a deep and detailed understanding of the factors influencing total mortality at the European country level, providing valuable information for the development of effective and targeted public health policies.

It emphasizes the need for an integrated and coordinated approach to tackle the complexity of health determinants and improve the health of the population at European level. The results of this study could help in the formulation of public health policies in European countries, i.e., policies for the prevention and treatment of chronic diseases; improving access to modern treatments; policies for the management and prevention of diabetes mellitus; and policies for nutrition and the promotion of healthy lifestyles. These policies need to be tailored to the specificities of each country, given the significant variability in health indicators between EU member states. Investments in health education, access to quality health care, and improved socio-economic conditions are important to achieve these objectives. The limitations of the research consist of the relatively small number of indicators used in the analysis and the limited time frame, which was 2010–2021. The analysis is unsuitable for predicting future occurrences or for extrapolating results to a broader population. Direct causality between combined evaluated determinants and death cannot be established.

Future research directions should further explore causal relationships and include additional variables that might influence total mortality. Specific interventions that have been shown to be effective in reducing mortality in some member states should also be investigated for replication in other regions.

## Figures and Tables

**Table 1:** Presentation of indicators.

No. Crt.	Indicator/Definition	Unit of Measurement	Symbol	Variable Type
1	Causes of death—standardized total mortality rate	Deaths per 100,000 inhabitants	ALLCD	Dependent
2	Standardized mortality rate for acute myocardial infarction	Deaths per 100,000 inhabitants	AMCINF	Independent
3	Total fat intake	Grams per capita per day	TFATS	Independent
4	Standardized mortality rate avoidable by prevention and treatment	Deaths per 100,000 inhabitants	AVMORT	Independent
5	Standardized mortality rate for diabetes mellitus	Deaths per 100,000 inhabitants	DIABMEL	Independent
6	Life expectancy at birth	years	LIFEEXP	Independent

Source: Realized by author according to OECD data.

**Table 2:** Hausman test.

Variable	Coefficients		Difference	Std. Err.
	(b) Fixed	(B) Random	(b-B)	
AMCINF	0.3428029	0.6555691	−0.3127662	0.1059765
TFATS	0.1341731	−0.1728298	0.3070029	0.0485576
AVMORT	1.654937	1.237987	0.4169493	0.0490104
DIABMEL	0.9845809	1.159471	−0.1748903	0.0059683
LIFEEXP	−15.83329	−23.14793	7.314632	0.2348565

b = Consistent under H0 and Ha; obtained from xtreg. B = Inconsistent under Ha, efficient under H0; obtained from xtreg. Test of H0: Difference in coefficients not systematic. Chi^2^ (5) = 124.06, Prob > chi^2^ = 0.0000. Source: Realized by the authors.

**Table 3:** Fixed-effects model.

Fixed-effects (within) regression			Number of obs = 300			
Group variable: DMU			Number of groups = 25			
R-squared:			Obs per group:			
Within = 0.8133			min = 12			
Between = 0.9409			avg = 12.0			
Overall = 0.9337			max = 12			
corr(u_i, Xb) = −0.3867			F (5270) = 235.30			
			Prob > F = 0.0000			
ALLCD	Coefficient	Std. err.	t	*p* > |t|	[95% conf. interval]	
AMCINF	0.342	0.215	1.59	0.112	−0.081	0.766
TFATS	0.134	0.187	0.72	0.475	−0.234	0.503
AVMORT	1.654	0.107	15.32	0.000	1.442	1.867
DIABMEL	0.984	0.233	4.21	0.000	0.524	1.444
LIFEEXP	−15.833	3.162	−5.01	0.000	−22.059	−9.606
_cons	1735.049	278.584	6.23	0.000	1186.575	2283.523
sigma_u 55.478						
sigma_e 22.287						
rho 0.86103569 (fraction of variance due to u_i)						

Source: Realized by the authors.

**Table 4:** Descriptive statistics.

Variable	Obs	Mean	Std. Dev.	Min	Max
ALLCD	300	961.4401	211.6277	670.7	1555.967
AMCINF	300	44.47205	18.41467	13.03571	112.6
TFATS	300	135.2651	19.51813	88.2	183.7
AVMORT	300	260.5853	106.5784	135.4	532
DIABMEL	300	20.0142	10.78941	7.9	103.56
LIFEEXP	300	79.45033	2.986911	71.4	84

Source: Realized by the authors.

## Data Availability

The data that support the findings of this study are available from the corresponding author upon request.
